# MCL Reconstruction Using a Flat Tendon Graft for Anteromedial and Posteromedial Instability

**DOI:** 10.1016/j.eats.2021.10.019

**Published:** 2022-02-08

**Authors:** Elisabeth Abermann, Guido Wierer, Mirco Herbort, Robert Smigielski, Christian Fink

**Affiliations:** a Gelenkpunkt Innsbruck, Austria; b Research Unit for Orthopaedic Sports Medicine and Injury Prevention, UMIT, Hall, Austria; c LKH Hall in Tirol, Hall, Austria; d OCM Clinic, Munich, Germany; e LIFE Institute, Warsaw, Poland

## Abstract

The main principles of the present medial collateral ligament (MCL) reconstruction techniques are (1) to approximate the natural anatomy and (2) to restore the main passive restraining structures in anteromedial and posteromedial knee instability. Therefore, we describe a technique using a flat tendon graft instead of tubular grafts with point-to-point bone fixation. Moreover, we address the deep MCL, a relevant restraint to anteromedial instability.

## Introduction

The medial collateral ligament (MCL) is the prime static stabilizer of the medial side of the knee joint. It is important for providing support against valgus stress, rotational forces, and anterior translational forces on the tibia. Injuries to that ligament are the most common injuries to the knee joint.^1^^,^^2^ Most of these injuries can be treated conservatively with good clinical results. However, reconstructions are required in major instability, chronic situations, and multifilament injuries.^3^ Persistent laxity results in increased load in the cruciate ligaments, which may cause early graft failure following cruciate ligament reconstruction.^4^, ^5^, ^6^, ^7^, ^8^, ^9^, ^10^, ^11^ Accordingly, biomechanical studies found that the superficial MCL (sMCL) is the major restraint to valgus rotation and external tibial rotation, especially in knee flexion. The posterior oblique ligament (POL) is an important restraint to internal tibial rotation and valgus rotation in full extension.^12^, ^13^, ^14^ Nevertheless, recent studies highlight the deep MCL (dMCL) relevance in controlling external tibial rotation.^13^^,^^15^, ^16^, ^17^ Moreover, the anterior part of the dMCL seems to be analogous to the ALL on the lateral side, perhaps an anteromedial ligament (AML), as was stated by Williams et al.^18^ Consequently, various MCL injury patterns, including the dMCL, sMCL, and POL might be observed.

**Fig 1:**
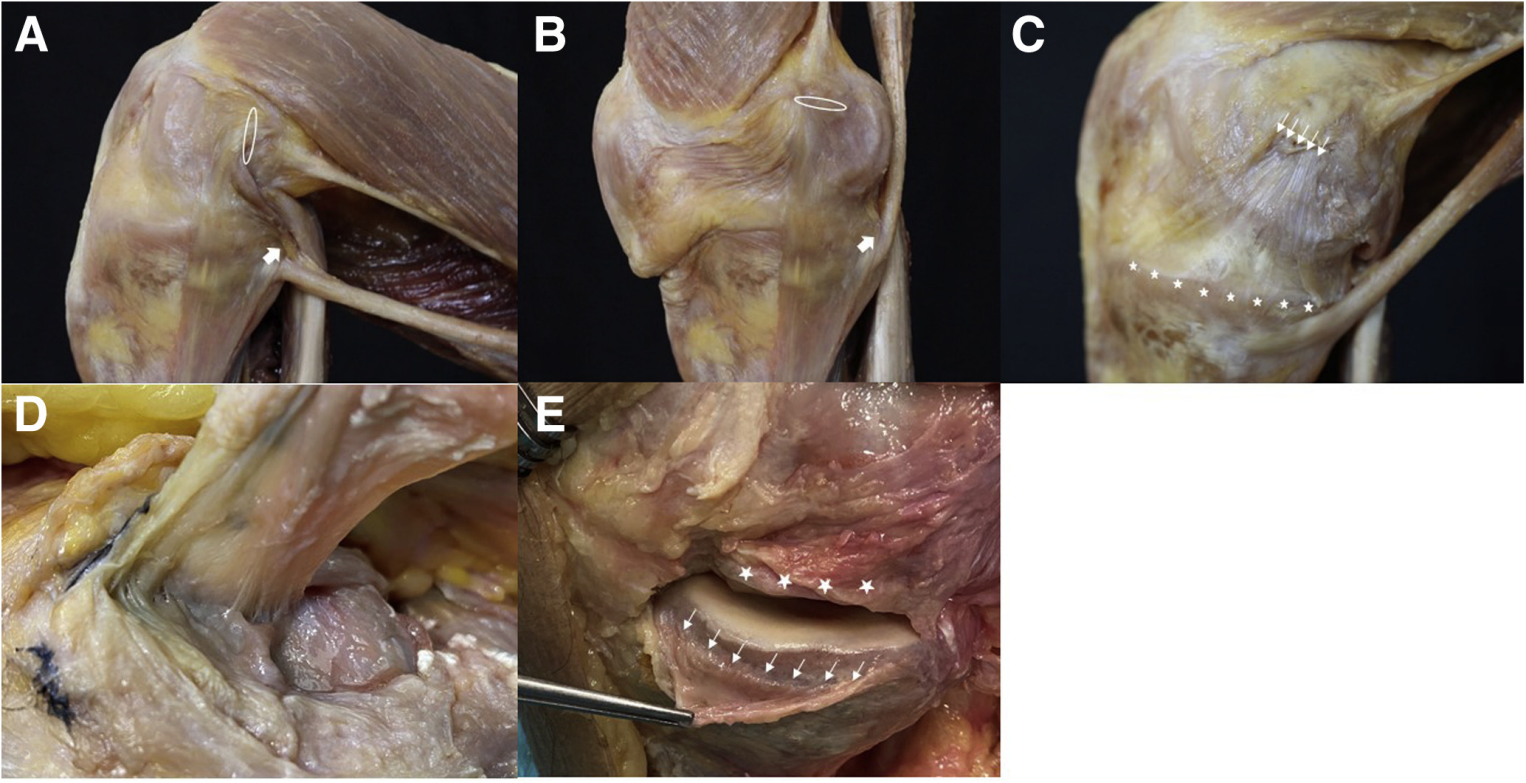
Anatomy of the superficial medial collateral ligament (sMCL) and deep medial collateral ligament (dMCL). As described by Liu et al.,^29^ the sMCL is broad and flat, composed of parallel and in the midpart oblique fibers with a wide insertion site on the medial femoral epicondyle^3^^,^^17^ (A) on a cadaveric right knee in about 110° of flexion seen from medial and the same knee in extension (B), the white oval showing the insertion site and its change of orientation due to flexion and extension and the arrow pointing at the tibial insertion of the posterior oblique ligament (POL). The direct inserting fiber are orientated in a narrow straight line (D) looking at the insertion site from below with the sMCL distally already detached and lifted. The dMCL fibers are fanning out distally from their femoral insertion site, which is a little bit posterior and distal to the sMCL insertion to a very wide tibial attachment from the PMC to ∼1 cm anterior to the anterior margin of the sMCL^3^^,^^17^ (C) on the same cadaveric knee with the sMCL completely detached, the small arrows marking the femoral insertion site of the sMCL and the asterisks showing the wide tibial insertion of the dMCL. (E) Tibial insertion marked by the small arrows about 8 mm below the joint line with the dMCL incised below the meniscus (asterisks).

**Fig 2:**
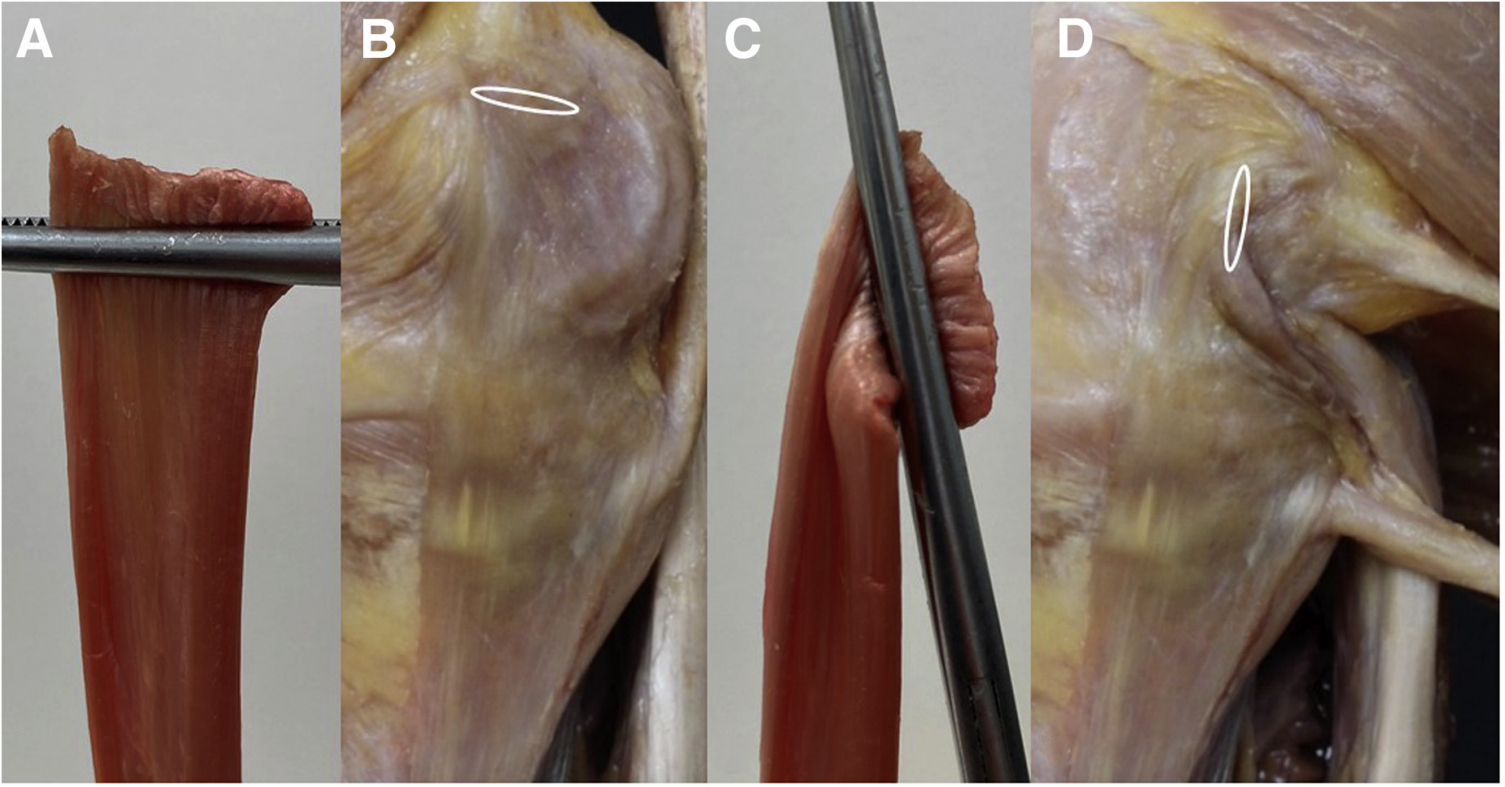
Appearance of a flat “ribbon-like” ligament in extension and flexion. A flat ligament is behaving differently than a round tendon. If a flat ligament has a wide insertion and not an insertion point, different fibers are under tension, depending on the position of the knee. (A) SemiT prepared to be a flat graft can be seen under tension with parallel end points like the superficial medial collateral ligament (sMCL) in a knee in extension (B). (C) The same graft can be seen with the upper fixation flexed almost 90°. (D) The appearance of the sMCL in a knee in flexion is shown.

Consequently, the MCL is a complex ligament to be reconstructed satisfactorily.^18^^,^^19^ Smigielski et al.^20^ described the anterior cruciate ligament’s flat, ribbon-like appearance. Similarly, the anatomy of the MCL is rather flat with broad insertion sites (Figs 1 and 2),^3^^,^^21^^,^^22^ and for the dMCL, an inverted fan shape passing from a small femoral attachment to a wide tibial attachment has been described (Fig 1, C and E).^15^ Therefore, we developed an anatomy resembling technique using a flat graft for reconstruction. Moreover, two MCL reconstruction techniques of the dMCL/sMCL and sMCL/POL are presented to address anteromedial and posteromedial knee laxities.

**Fig 3:**
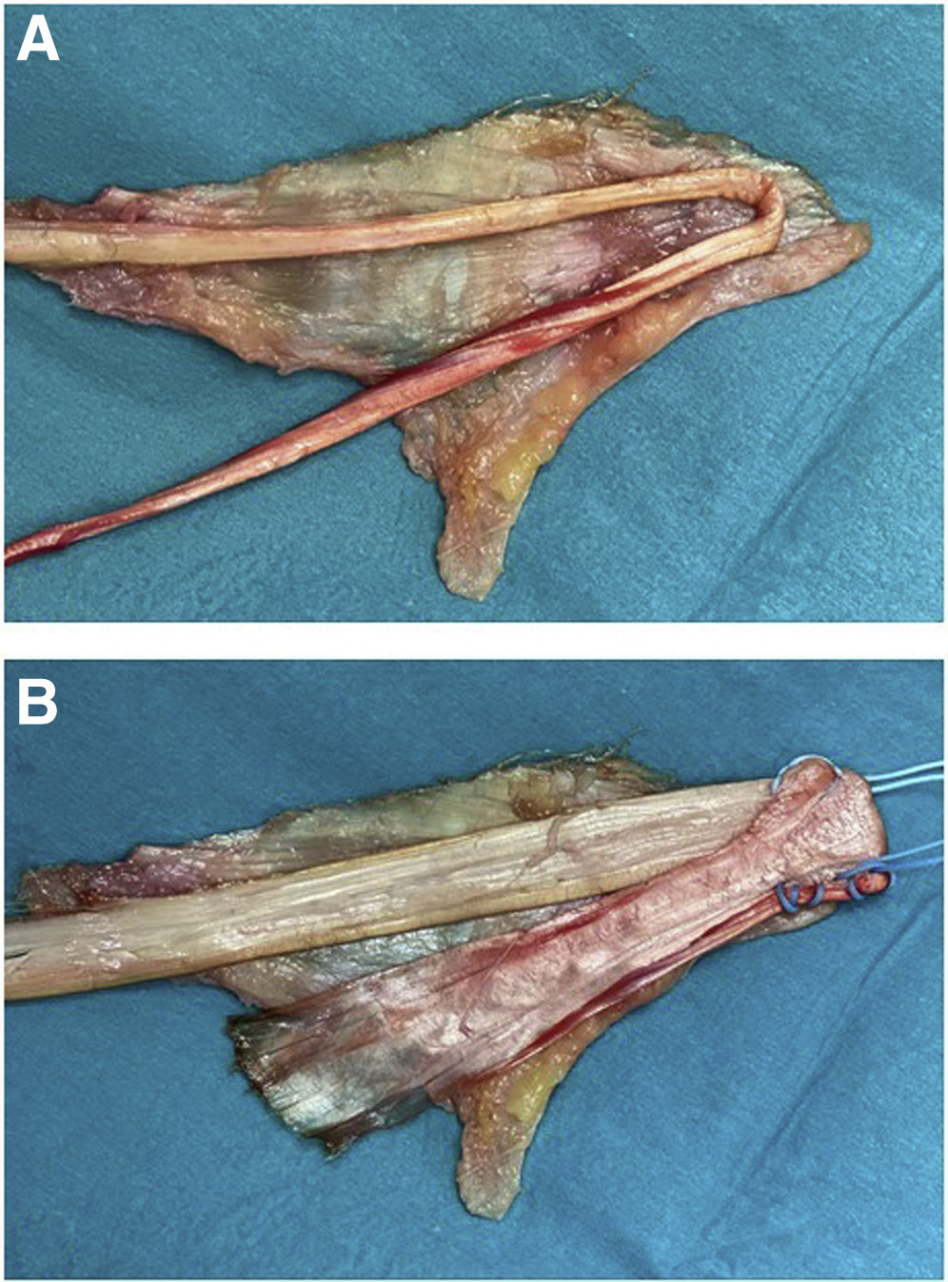
Tubular versus flat graft for medial collateral ligament reconstruction. The superficial medial collateral ligament (sMCL) and posterior oblique ligament (POL) are completely detached and put on the table with the POL part facing down. On top, either a tubular graft (A) or a flat graft (B) is put to show what would be reconstructed using each graft.^2^

**Fig 4:**
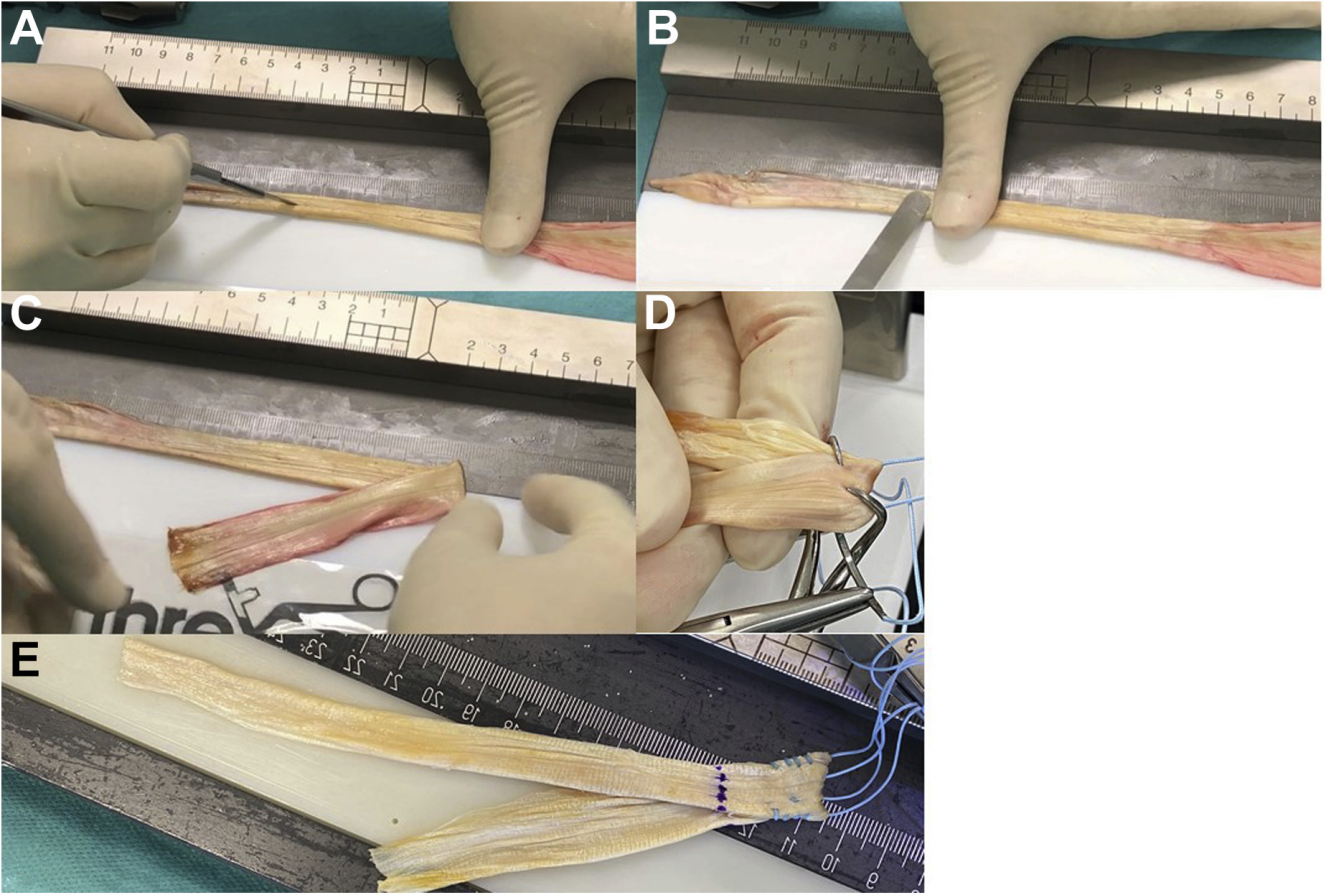
Graft preparation. The tendon is put on a preparation board, and any fatty or muscular tissue is already removed. The round tendon part of the semiT then is dissected with a knife to half of its diameter (A, with a semiT allograft) and subsequently smoothed into a flat shape by blunt raspatorium at minimum pressure (B), as already described by Fink et al.^30^ Domnick et al.^24^ showed this does not affect the structural properties of the tissue. After that, the graft is folded once in 1/3 to 2/3 at an angle of about 45° (C) over a suture sling that is removed after preparation and kept in place with grasping forceps (D). Then the folded end is prepared using an interlocking suturing technique with one or two—depending on the graft size—size 2 nonabsorbable sutures (FiberWire; Arthrex, Naples, FL), shown in D and E.

**Fig 5:**
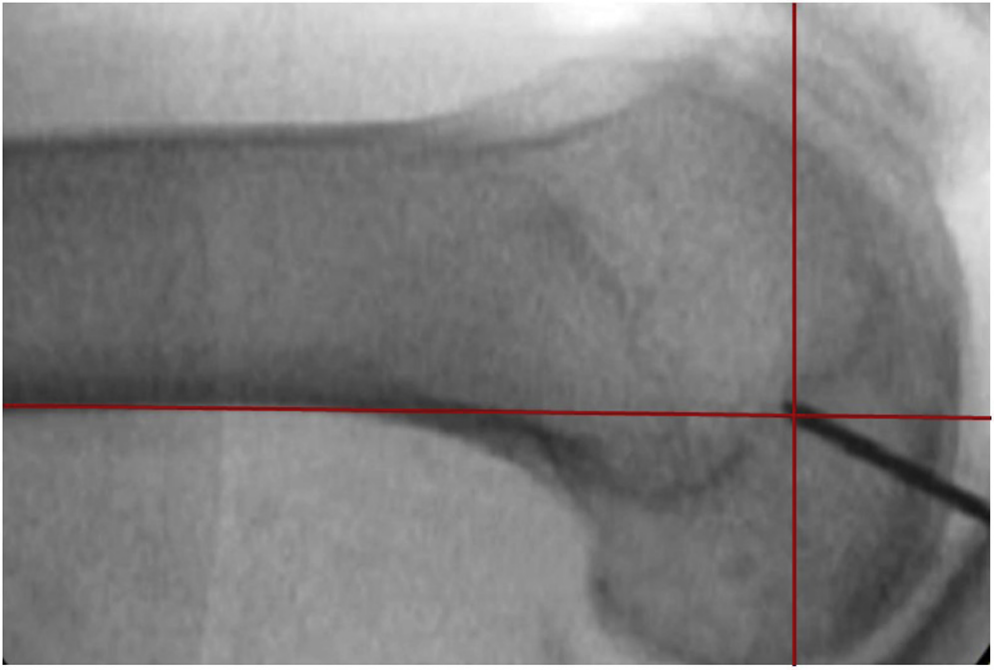
Radiographic landmarks for locating the femoral origin of the superficial medial collateral ligament (sMCL). A lateral view is shown of a right knee under fluoroscopy with a K-wire in the center of the femoral insertion of the sMCL. Line 1 is drawn parallel to the posterior aspect of the posterior femoral cortex (*y*-axis), and line 2 (*x*-axis) is drawn perpendicular to line 1, where line 1 intersects the Blumensaat line. The K-wire should be placed close to the intersection of the two lines in the proximal-anterior and proximal-posterior quadrant.^25^

**Fig 6:**
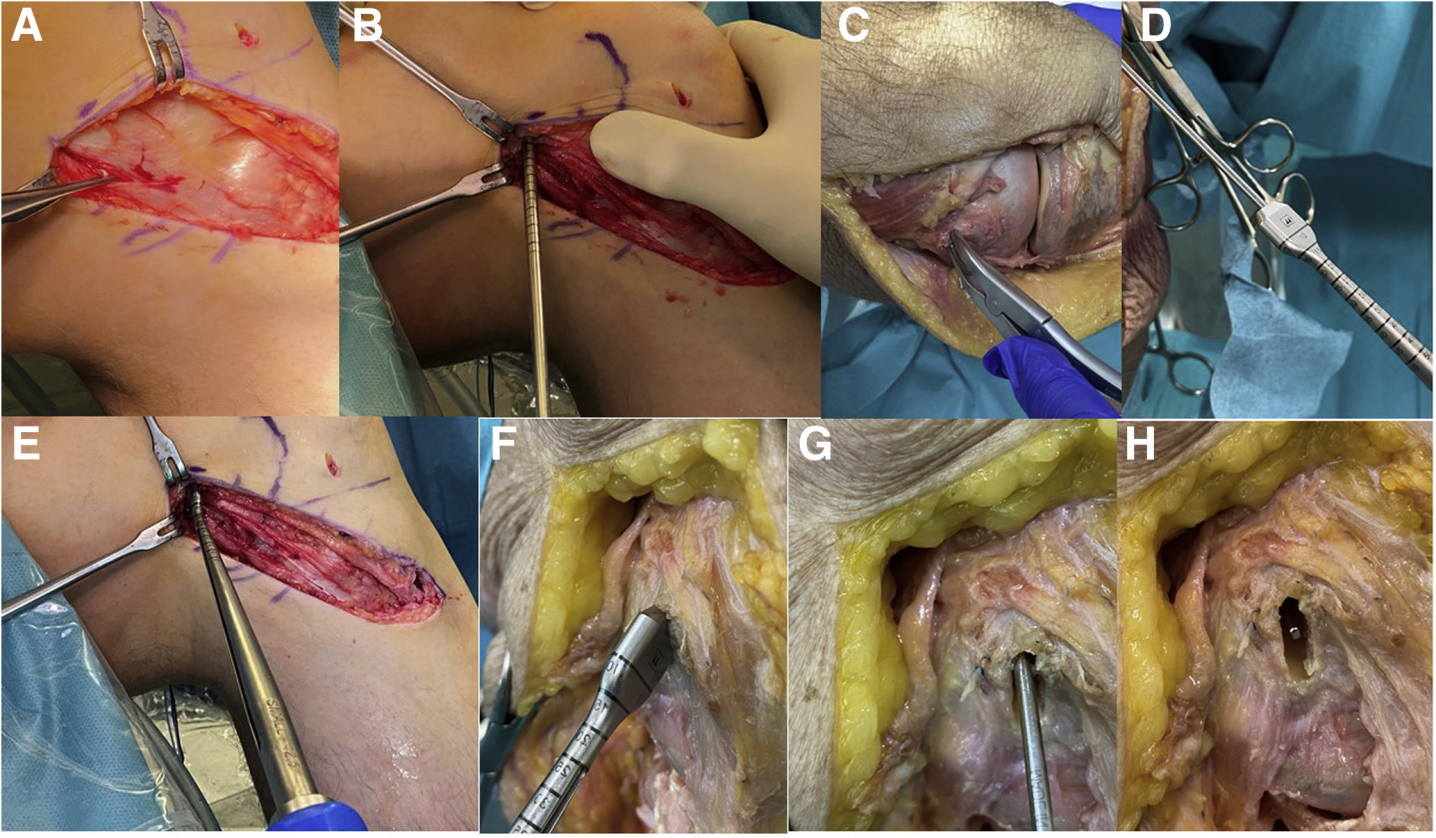
Aiming for a flat femoral tunnel. (A) A right knee can be seen from medial. The skin incision and subcutaneous preparation were already done. The forceps points to the medial epicondyle with the fascia still intact. At the level of the epicondyle, the fascia is opened lengthwise, a Beath Pin is inserted in the centre of the superficial medial collateral ligament (sMCL) insertion and overdrilled with a 4.5-mm drill bit to the lateral cortex (B). With the medial cortex in line with the insertion already removed with a luer (C), the flat dilator (Medacta International, Lugano, Switzerland) in the corresponding size; a small or medium dilator is inserted over the Beath Pin (D) and impacted to a depth of 25 mm into the medial condyle, as determined by the calibrations on the instrument (E and F). After removing the dilator (Medacta International, Lugano, Switzerland), a flat tunnel is established with the Beath Pin still in place (G and H).

### Surgical Technique

#### Positioning

The patient is positioned supine with the knee slightly flexed. Positioning should allow the patient to hang his or her leg down on the lateral side to perform at least a diagnostic arthroscopy and check for the reduction of the medial compartment. A thigh tourniquet is applied, and knee motion between 0° and 100° flexion should be possible. In this case, we prefer not to use a leg holder. For unlimited access to the operated leg’s medial side, the contralateral leg is put to lithotomy position.

#### Graft Harvesting

Autografts, including the semitendinosus tendon (SemiT) and the anterior half of the peroneus longus tendon, and various allografts can be used, according to the individual case and surgeon’s preference. However, the SemiT is an important dynamic stabilizer against valgus close to extension.^23^ It is essential to use a tendon with a flat muscle insertion. In case of an allograft, it is advised not to oversize the graft. Depending on the size of the knee, a graft of at least 22 to 26 cm is needed. In an acute case, in which a primary repair is performed, and augmentation is needed, an ipsilateral gracilis tendon left attached to the pes anserinus may be sufficient (Fig 3A).

#### Graft Preparation

1. The tendon length is checked on a preparation board, which should be 22 cm or more depending on the size of the knee.
2. The muscle is removed, and the tendon is placed with the muscular insertion facing upward.
3. At the end of the muscular insertion site, the tendon gets tubular. The tubular part of the tendon is flattened by dissecting the tendon to half of its diameter with a knife (Fig 4A) and smoothing it into a flat shape with a rasp (Fig 4B), as previously reported by Domnick et al.^24^ (Video 1).
4. The tendon is then folded at an angle of about 45° (Fig 4C) over a suture that is removed after preparation and kept in place with a Lahey-goiter grasping forceps (Fig 4D). Depending on the desired reconstruction (AML/sMCL or sMCL/POL), the anterior and posterior arm are folded either 1/3 to 2/3 or 2/3 to 1/3 of the graft length (Fig 3B, Video 1).
5. The folded end of the graft is prepared using an interlocking suturing technique (Krackow-stitch) with a no. 2 nonabsorbable suture (FiberWire; Arthrex, Naples, FL) and threaded as a pulley system into a fixation button (Fig 4E, Video 1).
6. The length of the femoral insertion (about 2 cm) should be marked on the graft.

#### Femoral Tunnel—Flat

1. A skin incision of about 12 cm in length is made from the medial epicondyle (Fig 6A) to the pes anserinus and open the sartorius fascia, so it can be sutured at the end of the procedure in the whole length.
2. A Beath Pin is drilled in the middle of the femoral insertion of the sMCL through the lateral cortex (Video 1). In case of a confusing anatomical situation after chronic injury to the medial structures, a lateral view picture with fluoroscopy can help to find the correct femoral insertion, according to Harthorn et al.^25^ (Fig 5).
3. Then the guidewire is over-reamed with a 4.5-mm drill bit through the lateral cortex (Fig 6B).
4. **Key Maneuver:** The knee is brought to full extension, and the tunnel orientation is marked parallel to the tibial plateau. Then the medial cortex is removed in the insertion area using a luer (Fig 6C). The flat dilator (Medacta International; Lugano, Switzerland) matching the graft size (small, medium, and large) is inserted over the guide wire (Fig 6D) and oriented parallel to the joint line in extension by tapping the back of the handle to a depth of 25 mm and then removed (Fig 6, F-H, Video 1).

#### Femoral Tunnel—Round

Alternatively, a round femoral tunnel is a good compromise if there is no time or a tunnel conflict due to complex ligament reconstructions. In this case, the sutures of the proximal end of the graft are inserted in the Beath Pin without a fixation button.

1. A Beath Pin is drilled in the femoral insertion of the sMCL through the lateral cortex.
2. Then the guidewire is overdrilled with at least an 8 mm drill bit or matching the graft size to a depth of 30 mm and removed (Video 1).

### Superficial MCL and deep MCL/AML Reconstruction

#### Graft Insertion

1. The Beath Pin is used to pass the lead sutures and the pulley sutures of the femoral button through the soft tissue proximo-laterally.
Alternatively, in case of a round tunnel, the lead sutures of the graft are pulled out proximolaterally. The graft is not yet inserted into the tunnel (Video 1).
2. The fixation button is introduced by pulling on the lead sutures until flipping outside the lateral cortex.
3. Now the pulley sutures are tightened, and the graft is inserted to the entrance of the femoral tunnel with the short arm being below and facing anterior and the long arm being superficial and facing posterior (Fig. 7A, B).

**Fig 7:**
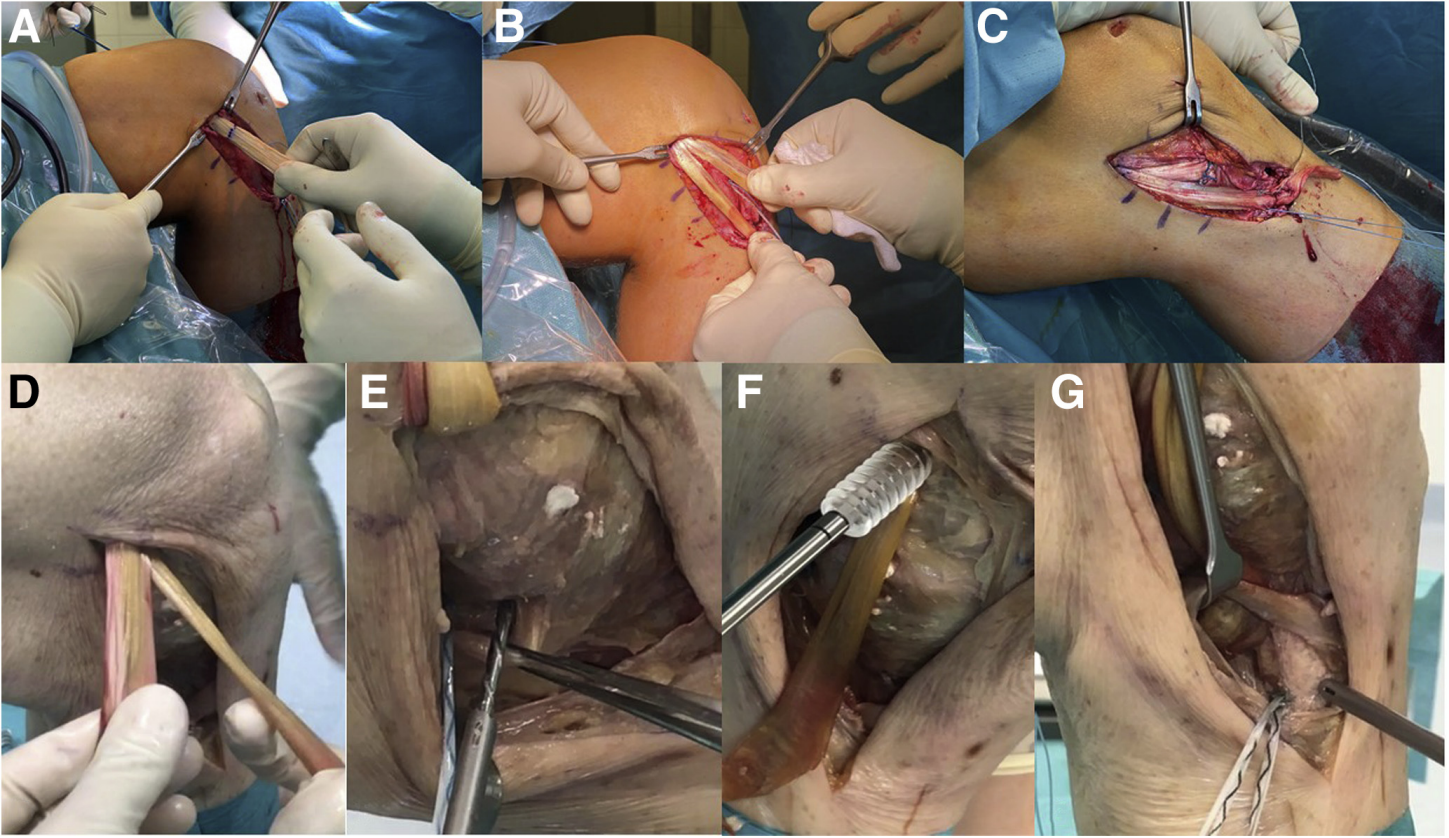
Graft insertion is shown on a left knee. To insert the graft, the lead sutures of the fixation button and the pulley sutures are passed with the Beath Pin through the femoral tunnel and the soft tissue proximolaterally, and the graft is entered in the femoral tunnel, ensuring the right orientation of the graft (A). The graft is inserted into the tunnel almost to the marking, but not yet fixed, and the two arms are checked for their length (B) before—in case of a superficial medial collateral ligament (sMCL)/anteromedial ligament (AML) reconstruction—the anterior shorter arm is fixed to the tibia with two suture anchors (e.g., 2.6 FiberTak; Anchors, Arthrex, Naples, FL) at the appropriate length. Then the femoral button is fixed under tension near to extension and in neutral rotation, and the long arm is fixed to the tibial insertion area of the sMCL beneath the Pes anserinus (C). In case of a sMCL/posterior oblique ligament (POL) reconstruction, the graft is inserted in the opposite direction with the short arm aiming posteriorly (D). That arm is then fixed to the tibial insertion of the POL, adjacent to the semimembranosus tendon, and posterolaterally to it with two suture anchors (E). Afterward, the graft is fully pulled into the femoral tunnel with the knee in extension and in a neutral rotation. In this reconstruction, the alternative using a round femoral tunnel with screw fixation is shown (F). Then the sMCL is fixed to its tibial insertion area with two suture anchors under tension close to extension (G).
4. For tibial fixation of the dMCL or AML, a suture anchor is placed about 8 mm distally to the joint line and anteriorly to the sMCL. A second one is placed about 1 cm anterior to the first anchor to reconstruct the fibres limiting external rotation (Fig 7C, Video 1).
5. The anterior arm of the graft is tied to the suture anchors without any tension.
6. Next, the knee is brought to almost full extension and neutral rotation and the pulley sutures are tightened until the dMCL is under tension and tied over the fixation button with a knot-pusher.
Alternatively, for the round tunnel, the graft is tightened in almost full extension and neutral rotation and fixed with a fully threaded cannulated bio-absorbable interference screw. The screw is inserted over a guidewire proximal to the graft matching tunnel diameter (Fig 7F, Video 1).
7. The deep arm is sutured distally to the posterior end of the femoral tunnel to the soft tissue remnants of the deep MCL with absorbable sutures.
8. In 20° of knee flexion, the distal insertion of the sMCL is exposed below the Pes anserinus, and two suture anchors are inserted in the insertion area – one on the posterior border and one on the anterior border (Fig 7G).
9. The posterior and long arm of the graft is now tied to the suture anchors under tension.
10. The two margins of the long arm of the graft are sutured with absorbable sutures to the remaining soft tissue about 15 mm distal to the joint line.
11. Now the knee is checked for unrestricted range of motion from full extension to at least 100° of knee flexion. The dMCL should tighten in external rotation and the sMCL in slight flexion (Fig 8A, Video 1). In full extension only, the POL is under tension.

**Fig 8:**
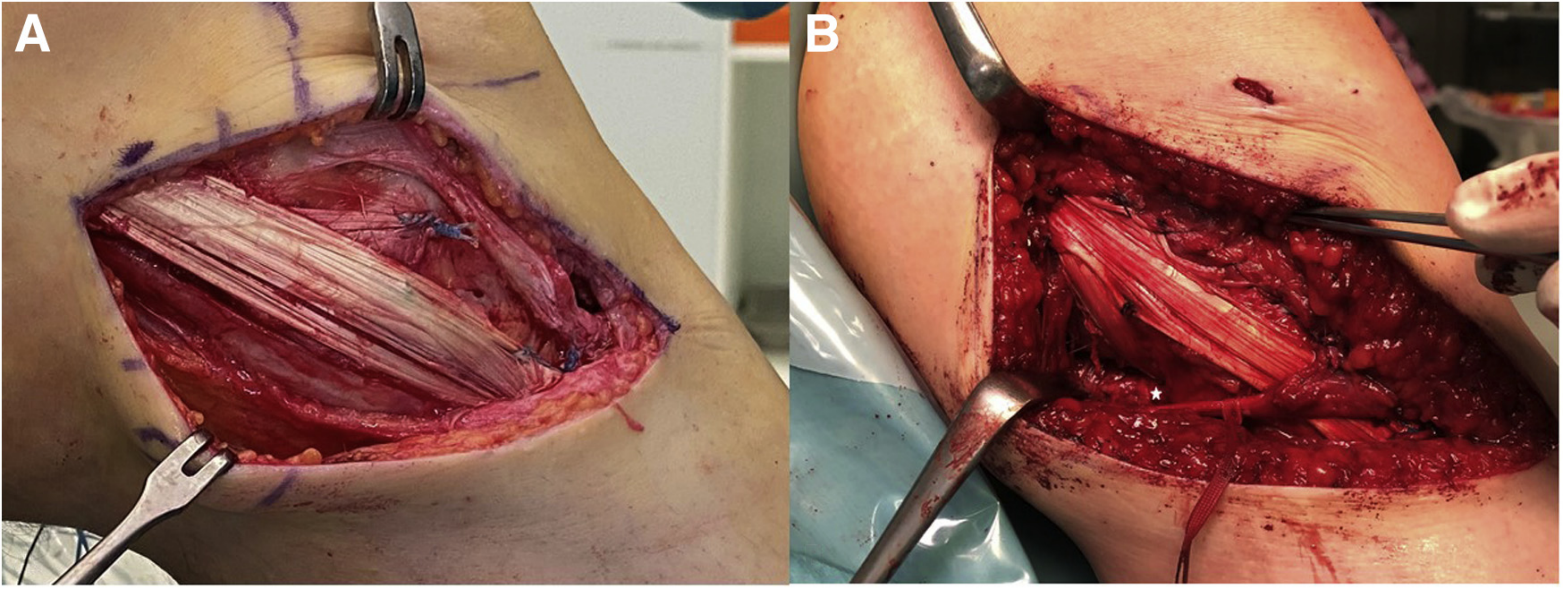
Final graft appearance. (A) The final graft appearance is seen of a superficial medial collateral ligament (sMCL)/anteromedial ligament (AML) reconstruction using a semiT allograft in a left knee with anteromedial instability in an anterior cruciate ligament (ACL) revision case from a medial view. The pes anserinus already used for the ACL graft in the primary reconstruction partially and, therefore, missing. (B) Final situation of a sMCL/posterior oblique ligament (POL) reconstruction using a semiT autograft in a left knee with posteromedial instability. The gracilis tendon is still in place and crosses the sMCL. The white asterisk marks the semimembranosus tendon adjacent to the tibial insertion of the POL.
12. Finally, the reduction of the medial compartment should be documented under arthroscopy or image intensifier (Fig 9), and the sartorius fascia should be closed for additional medial stability.

**Fig 9:**
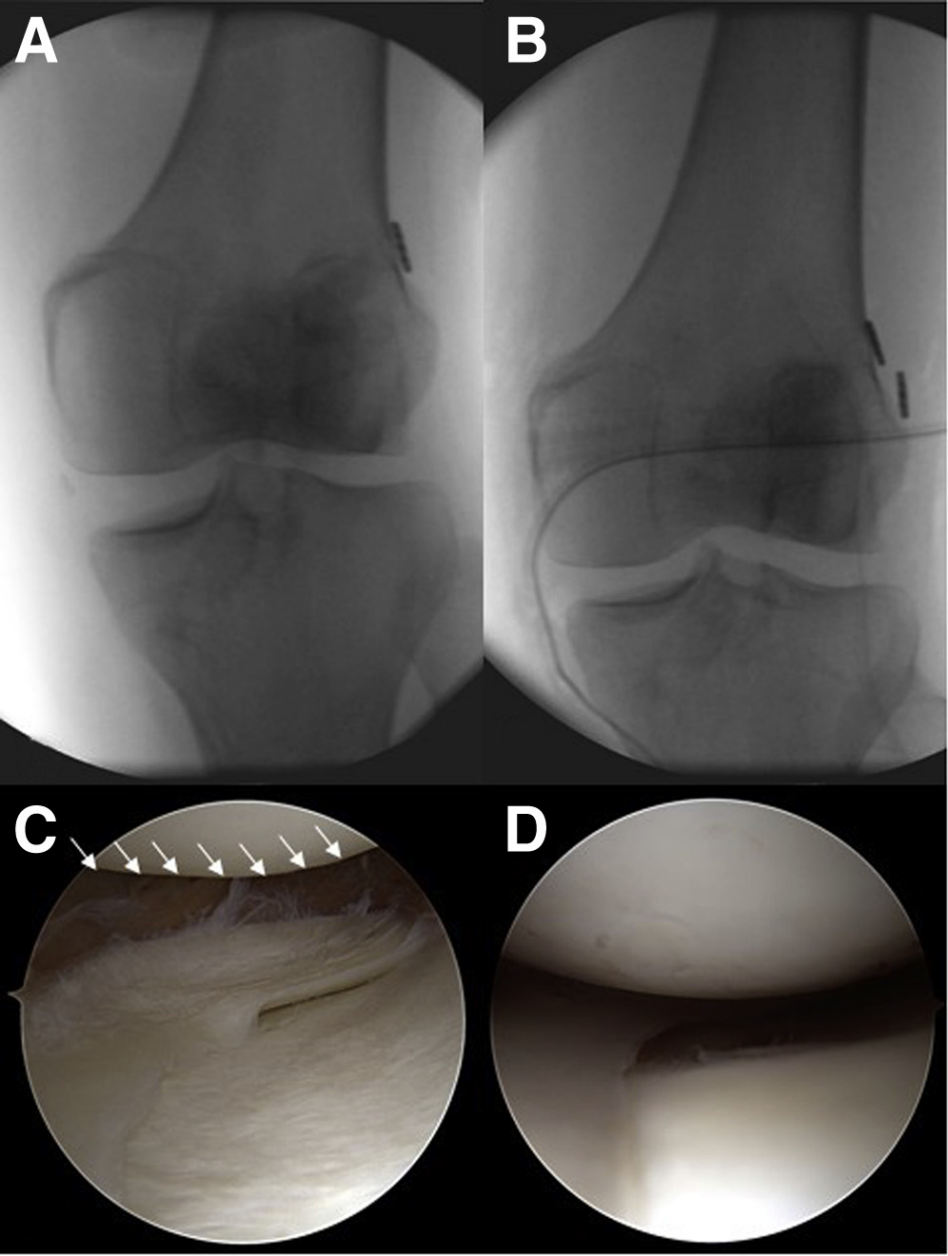
Preoperative and postoperative imaging of medial laxity. (A) An ap-view of a left knee with chronic medial instability after anterior cruciate ligament revision surgery. The medial laxity under valgus stress is obvious under image intensifier. (C) The corresponding arthroscopic image with a medial drive-through sign is presented from a lateral portal view (asterisks show the lift off of the femoral condyle). (B and D) Situation after medial collateral ligament reconstruction.

### Superficial MCL and POL Reconstruction

#### Graft Insertion

1. The Beath Pin is used to pass the lead sutures and the pulley sutures of the femoral button out through the femoral tunnel and the soft tissue proximolaterally.
Alternatively, in case of a round tunnel, the lead sutures of the graft are pulled out proximolaterally, and the graft is not yet inserted into the tunnel (Video 1).
1. The fixation button is introduced by pulling on the lead sutures until flipping outside the lateral cortex.
2. Now the pulley sutures are tightened, and the graft is inserted into the entrance of the femoral tunnel with the short arm being below and facing posterior and the long arm superficial and facing anterior (Fig 7A).
3. For the POL’s tibial fixation, the semimembranosus tendon is carefully exposed (Fig 1A), and a suture anchor is inserted straight proximally to it (Fig 6E), and a second one is inserted1 cm posterolateral to it at the same level.
4. Then the posterior arm of the graft is tied to the suture anchors without any tension. Any remnants of the native POL can be sutured to the same anchors.
5. Next, the knee is brought to full extension, and neutral rotation and the pulley sutures are tightened until the POL is under tension and tied over the fixation button with a knot-pusher (Video 1).
Alternatively, for the round tunnel, the graft is tightened in full extension and neutral rotation and fixed with a fully threaded cannulated bioabsorbable interference screw. The screw is inserted over a guidewire proximal to the graft-matching tunnel diameter (Fig 7F).
6. In 20° of knee flexion, the distal insertion of the sMCL is exposed below the Pes anserinus, and two suture anchors are inserted in the insertion area: one on the posterior border and one on the anterior border (Fig 7G).
7. The anterior and long arm of the graft is now tied to the suture anchors under tension.
8. The anterior and posterior margins of the long arm of the graft are sutured with absorbable sutures about 15 mm distal to the joint line to the remaining soft tissue.
9. The knee is fully extended and flexed at least to 100° to check for unrestricted range of motion. The POL should be only tight in extension and the superficial MCL in light flexion (Fig 8B, Video 1).
10. Finally, the reduction of the medial compartment should be documented under arthroscopy or image intensifier (Fig 9), and the sartorius fascia should be closed for additional medial stability.

#### Postoperative Care

A hinged knee brace limiting range of motion 0° to 90° is applied for 6 weeks, if no additional injuries to the posterior ligamentous structures and no meniscal injuries are present. Mobilization starts with partial weight-bearing of 15 to 20 kg for 2 weeks. Active range-of-motion exercises are initiated immediately, focusing on gaining full extension after the second week. Full weight-bearing can be commenced thereafter as tolerated if no additional injuries are apparent. Physical therapy is recommended right after the hospital stay 2 to 3 times per week for at least 8 to 12 weeks or free range of motion and adequate muscular rehabilitation.

## Discussion

We describe a versatile reconstruction technique for the medial side of the knee in either type of instability pattern using a flat “ribbon-like” graft to be closer to native MCL anatomy compared with commonly used techniques with tubular graft types. Additionally, to anatomical superiority, usage of flat grafts provides other advantages: “Reshaping” a tendon graft with a round cross-sectional area (e.g., hamstring tendon) to a flat graft by dissecting the tendon to the half of its diameter with a knife, and smoothing it to a flat shape is technically feasible. According to Domnick et al.,^24^ this preparation was also found not to affect the biomechanical properties of the graft. The bone contact area of a flat graft is greater compared to a round one, which showed a positive biological effect on tendon-to-bone healing in a recent animal study.^26^ Furthermore, the flat graft geometry is able to reproduce the native MCL appearance with wide insertion areas on the femoral and tibial side^3^^,^^17^ better than a tubular graft used in conventional reconstruction techniques.^27^^,^^28^ Another aspect in favor of a flat tendon graft is that fixation angles seem less critical in reconstruction techniques compared to tubular grafts. The latter resulting in “point-to-point” fixation, whereas in flat grafts, tension patterns change within the graft at different angles (Fig 2). Advantages and disadvantages comparing the flat tendon technique to a conventional technique are listed in Table 1. Pearls and pitfalls are described in Table 2.

**Table 1:** Advantages and Disadvantages

Technical Aspect	Advantages	Neutral	Disadvantages
Flat “ribbon like” graft	Recreates native fiber arrangement with either a hamstring or peroneus split autograft or a similar tendon allograft	No difference in graft size (cross-sectional area)	Learning curve for graft preparation
Flat femoral tunnel	Recreates anatomic MCL origin Better tendon to bone healing due greater contact area	Guidewire location is the same as conventional reconstruction techniques.	More surgical steps in tunnel preparation
Wide tibial insertion	Recreates a more anatomy resembling fiber orientation both in the AML and POL reconstruction		More suture anchors are needed—and, therefore, higher costs
Graft insertion	Graft orientation during insertion is obvious if preparation was correct		Graft orientation needs to be determined during preparation and cannot be corrected while inserted

AML, anteromedial ligament; MCL, medial collateral ligament; POL, posterior oblique ligament.

**Table 2:** Pearls and Pitfalls

Surgical Steps	Pearls and Pitfalls
Graft preparation	Make sure you have pulled strands in every portion of the proximal graft end; otherwise, you will struggle to get the midportion in the femoral tunnel.
Femoral flat tunnel	Fully extend the leg for orientation of the femoral tunnel and remove the cortex with a luer to facilitate the insertion of the flat dilator.
Tibial fixation AML	The tibial insertion area of the dMCL is very wide. In case of anteromedial instability, the most important fibers are the anterior ones to resist external rotation. Therefore, it is important to put the suture anchors anterior to the sMCL.
Tibial fixation POL	If the knee is not well centered, it is easy to fix the POL to far anterior, so the best option to find the right insertion is to expose the insertion of the semimembranosus and set the first suture anchor immediately proximal to it—usually, as small bursa can be found in this area—and the second one, even more posterolateral.
Graft insertion	Mark the estimated insertion depth on the graft to make sure you have enough bone tendon contact for healing and ensure the tunnel is long enough for some minor length adjustments.
Tibial fixation sMCL	The bone in the insertion area is already quite hard and cortical. Therefore, especially in young patients, it is better to drill a hole before using the obturator so that it can be removed easily.

dMCL, deep medial collateral ligament; POL, posterior oblique ligament; sMCL, superficial medial collateral ligament.

For graft choice in MCL reconstruction, one should take into account that the hamstring tendons and especially the semitendinosus tendon are important dynamic stabilizers against valgus rotation close to extension,^23^ and, therefore, an ipsilateral hamstring tendon graft may not be the best choice in a MCL deficient knee. Alternatively, contralateral hamstring grafts or a peroneus split graft as an autograft or several allografts can be used.

The most decisive limitation of this technique is the number of anchors needed to reproduce the wide insertion areas, which adds costs, the increased surgical time, and the need for special instrumentations for creating a rectangular tunnel. Especially in complex ligament surgery when time becomes an issue, as a compromise, the rectangular tunnel on the femur may be replaced by a conventional round tunnel using a cannulated drill and interference screw fixation still using a flat graft.

### Conclusion

The flat reconstruction technique for medial instability proved feasible and effective clinically and may be associated with biomechanical and biological advantages to the commonly used techniques with tubular grafts.

## Supplementary Data

ICMJE author disclosure forms

Video 1 This video shows two options of double-strand medial collateral ligament (MCL) reconstruction techniques to either perform an anteromedial or posteromedial stabilization using a flat graft with a thin oval-shaped insertion on the femur, a wide insertion of the oblique fibers close to the tibial plateau, and a large distal insertion of the superficial medial collateral ligament (sMCL) part, at the level of the pes anserinus. This reconstruction technique can be performed using autografts like semitendinosus tendon or peroneus split graft or allografts like semitendinosus tendon, peroneus longus tendon, or tibialis posterior tendon. “Round” tendons can be easily converted to a flat shape by dissecting the graft to half of its diameter and afterward squeezing it with a blunt rasp without any negative impact on mechanical properties of the graft. The graft is then folded once in 1/3 to 2/3 at an angle of about 45°, with the short arm being below and looking either anterior or posterior, depending on the aim of reconstruction. After suture fixation of the proximal end using Krackow stitches, the graft shows a permanent angle and flat shape. After exposing the femoral insertion of the MCL, a Beath Pin is drilled in the center of the insertion area through the lateral cortex. After overdrilling the k-wire and removing the medial cortex with a luer, the flat femoral socket parallel to the joint line in extension is established using a flat dilator (Medacta International; Lugano, Switzerland) matching the graft size (S.M). Alternatively, a round femoral tunnel is acceptable, especially in complex reconstructions and lack of time. In this case, the Beath Pin is positioned slightly more posterior in the MCL insertion area and overdrilled with a drill bit matching the graft size to a depth of 30 mm. The graft is inserted with the correct orientation of the two arms into the femoral tunnel, but not yet fixed. First, the short arm is attached to the tibia, either in the anteromedial ligament (AML) or posterior oblique ligament (POL) position using two suture anchors (e.g., 2.6 FiberTak Anchors; Arthrex, Naples, FL). Then the graft is fixed under tension in the femoral tunnel, either using the fixation button for the flat tunnel or a screw for the round tunnel. Finally, the long arm is fixed as sMCL to the tibia at the level of the pes anserinus, again using two suture anchors close to extension. Now, the knee is fully extended and is flexed at least to 100° to check for free range of motion.
